# Investigation of crystalline lens overshooting: *ex vivo* experiment and optomechanical simulation results

**DOI:** 10.3389/fbioe.2024.1348774

**Published:** 2024-04-09

**Authors:** Ali Dahaghin, Milad Salimibani, Agnieszka Boszczyk, Agnieszka Jóźwik, Marta Skrok, Jorge Grasa, Damian Siedlecki

**Affiliations:** ^1^ Department of Optics and Photonics, Wroclaw University of Science and Technology, Wrocław, Poland; ^2^ Aragón Institute of Engineering Research (I3A), University of Zaragoza, Zaragoza, Spain; ^3^ Centro de Investigación Biomédica en Red en Bioingeniería, Biomateriales y Nanomedicina (CIBER-BBN), Zaragoza, Spain

**Keywords:** porcine crystalline lens, inertial motion, wobbling, overshooting, finite-element method, fluid–structure interaction, Purkinje imaging

## Abstract

**Introduction:** Crystalline lens overshooting refers to a situation in which the lens momentarily shifts too much from its typical location immediately after stopping the rotational movement of the eye globe. This movement can be observed using an optical technique called Purkinje imaging.

**Methods:** In this work, an experimental setup was designed to reproduce this effect *ex vivo* using a fresh porcine eye. The sample was rotated 90° around its centroid using a high-velocity rotation stage, and the Purkinje image sequences were recorded, allowing us to quantify the overshooting effect. The numerical part of the study consisted of developing a computational model of the eye, based on the finite element method, that allowed us to understand the biomechanical behavior of the different tissues in this dynamic scenario. A 2D fluid–structure interaction model of the porcine eye globe, considering both the solid parts and humors, was created to reproduce the experimental outcomes.

**Results:** Outputs of the simulation were analyzed using an optical simulation software package to assess whether the mechanical model behaves optically like the real *ex vivo* eye. The simulation predicted the experimental results by carefully adjusting the mechanical properties of the zonular fibers and the damping factor.

**Conclusion:** This study effectively demonstrates the importance of characterizing the dynamic mechanical properties of the eye tissues to properly comprehend and predict the overshooting effect.

## 1 Introduction

Humans rely strongly on their eyes as one of their most critical sensory organs; therefore, accurate knowledge about the biomechanics and optics of the eyes is of particular importance as they might be exposed to various injuries and diseases. One of the most interesting mechanical phenomena that can be observed in the anterior part of the eye is the so-called lens wobbling or overshooting. This effect could be described as the oscillatory inertial motion of the crystalline lens, resulting from a rapid saccadic movement of the whole eyeball or change in the gaze direction. The inertial motion is a consequence of the rapid rotation and abrupt halt at which the lens, suspended by the ciliary body, is subjected. After the first qualitative observation of the lens wobbling phenomenon by D'Ombrain (1936) and then by Bartholomew (1970), a quantitative description was presented by Jacobi and Jagger (1981) while investigating intraocular lens (IOL) wobbling in pseudophakic eyes.

From the anterior part of the eye, Purkinje images (P) can be observed as an optical representation of eyeball movement to evaluate the oscillations of the crystalline lens. In general, these images come from specular reflections of light from the anterior and posterior surfaces of both the cornea and crystalline lens, respectively. In the case of a natural (healthy) crystalline lens, the Purkinje imaging technique is usually restricted only to the reflection from the posterior surface of the lens (Tabernero and Artal, 2014). Taking into account this limitation, studying the wobbling magnitude is an emerging, non-trivial topic. Only a few papers on the realistic display of finite element-based biomechanical simulations have been published so far (Stocchino et al., 2007; Martin et al., 2009). On the other hand, many eye structures have previously been modeled using analytical techniques or FEM to demonstrate other natural processes, for instance, the accommodation of the lens or the effects of elevated intraocular pressure on ocular structures (Lanchares et al., 2012; Ayyalasomayajula et al., 2016; Dai et al., 2017; Wang and Pierscionek, 2019; Cabeza-Gil et al., 2021a; Cabeza-Gil et al., 2021b; Shahiri et al., 2023). In a recent study by Boszczyk et al., it was assumed that wobbling consists of two motions, lateral displacement (decentration) and tilt, which significantly contribute to both captured and simulated Purkinje images of the human eye (Boszczyk et al., 2023). The same behavior was represented by a mechanical model developed for an intraocular lens implant (Martin et al., 2009). However, to the best of our knowledge, it has never been investigated by *ex vivo* studies, neither experimental nor numerical ones.

Numerical studies in the field of biomechanics often focus on understanding the complex interactions between fluids and structures within biological systems, because this kind of interaction plays a crucial role in various biological processes and medical applications, such as biomechanics of soft tissues, drug delivery, and eye diseases (Fernández-Vigo et al., 2018; Redaelli et al., 2022). Several studies have been conducted on the corneal, scleral, zonular, lens, and vitreous materials (Kling et al., 2014; Nejad et al., 2014), and the majority of them focus on understanding how different factors, such as variations in material properties and physiological and environmental factors (e.g., intraocular pressure), impact the mechanical behavior of the eye. The biomechanics of the eye seems to be substantially influenced by a variety of parameters, challenging prior findings (Ayyalasomayajula et al., 2016). Most of the referred studies aimed at static lens modeling rather than modeling the dynamic phenomena, with remarkable exceptions intended to simulate, i.e., the dynamic air-puff tonometry, saccadic and scattering (Ritschel et al., 2009; Abouali et al., 2012; Nishiyama et al., 2013).

The most critical stage of the modeling and simulation procedure is the selection of mechanical and geometric parameters of ocular tissues. Since some earlier studies demonstrated that material features may significantly influence the results of simulations (Bocskai and Bojtár, 2013), the behavior of the zonular fibers, vitreous body, and aqueous humor may be of particular importance in biomechanical simulations. Furthermore, the position of the lens during saccades, acceleration, and velocity should be monitored. The findings show that immediately after the saccadic motion, the vitreous body still undergoes some fluctuations (Martin et al., 2009). The complex and yet unknown interplay of all these factors makes ocular dynamics a subject of numerous clinical studies and investigations. The continuous development of technologies and instrumentation for estimating ocular dynamics remarkably supports the increasing interest in this research area.

Modeling of the porcine eye *ex vivo* is of particular interest for some reasons. It might be challenging to extract actual intraocular pressure (IOP) data or change other parameters such as velocity in *in vivo* tests. The general objective of this work is to broaden our knowledge of the dynamical behavior of the crystalline lens induced by rapid eye rotation, and it is expected to contribute to our understanding of the wobbling phenomenon. Based on the literature review from an optomechanical perspective, the characteristics of this lens motion have undergone limited investigation so far. It is likely that the inertial force on the eye impacts the internal tissues of the eye. Therefore, the quantification of these movements in the lens becomes fundamental. Meanwhile, the change in intraocular pressure may also have an effect. For this reason, this study is focused on finding a valid *ex vivo* optomechanical model and developing a computational model able to mimic it, which could be used for future numerical studies.

## 2 Materials and methods

### 2.1 *Ex vivo* experiment

**FIGURE 1:**
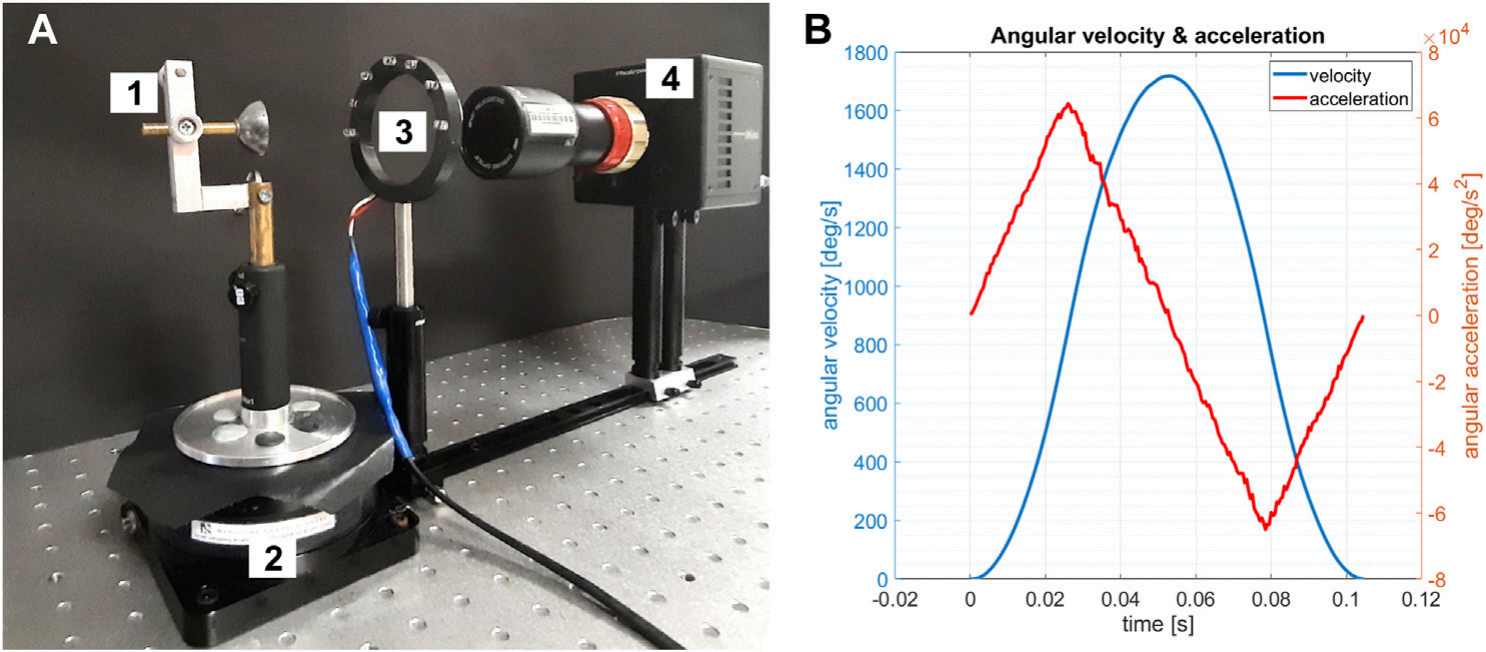
**(A)** Experimental setup: 1—porcine eye holder; 2—high-speed rotational stage with controlled angular velocity and acceleration; 3—semicircular illuminator; 4—high-resolution camera with a 0.5× telecentric lens. **(B)** Actual (measured) data of angular velocity (blue) and acceleration (red).

The first step in this study was to capture the performance of the Purkinje images due to the eye rotation. A porcine eye obtained from a local slaughterhouse (1.5–4.5 h *post mortem*) was examined on a custom-made experimental setup (Figure 1A), which consisted of an eye holder mounted to a high-speed precision rotation stage (8MRL120-15, Standa Ltd.) with controlled angular velocity and acceleration. A semicircular illuminator consisting of 7 infrared diodes (dominant wavelength of 850 nm) was placed approximately 12 cm in front of the porcine eye. The eye was tightly sewn to the holder with a surgical thread. The eye was rotated by 90° around a vertical axis toward the illuminator and a camera with a peak angular velocity and acceleration/deceleration of 1,700 deg/s and 60,000 deg/s^2^, respectively (see Figure 1B). These parameters were selected in order to meet the order of magnitude of the rotational acceleration of the human eye (Zhang et al., 2012). The Purkinje images were captured using a high-resolution camera (FASTCAM Mini UX50, Photron Limited) with a frame size of 1,280 × 1,024 pixels, pixel size of 10 × 10 μm, and frame rate of 640 fps. The camera was equipped with a 0.5× telecentric lens (GoldTL™ Telecentric Lens, Edmund Optics).​ An exemplary video sequence of the motion of the recorded Purkinje images is presented in Supplementary Video 1. Five similar sequences were captured and analyzed for the examined porcine eye.

**FIGURE 2:**
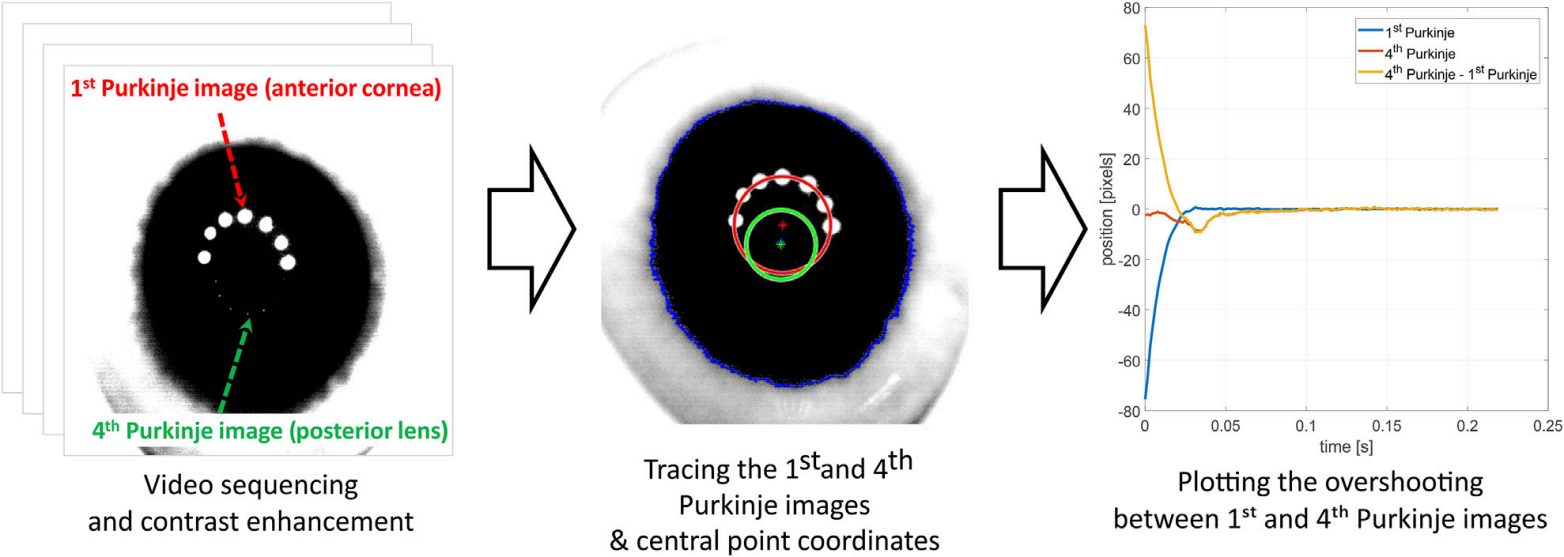
Experimental data processing scheme.

In order to trace the displacements of the Purkinje images, image processing procedures in MATLAB were applied. At each video frame, two circles were fitted to the light reflections from the eye—one representing the first Purkinje image and the second representing the fourth Purkinje image. The geometrical centers of these circles were recognized as P1 and P4 locations, respectively. For further analysis, the difference between the horizontal position of the P4 and P1 was calculated, which allowed us to focus on changes in the position of the lens relative to the whole eye. The scheme of experimental data processing is presented in Figure 2.

### 2.2 Mechanical simulations

#### 2.2.1 Geometry

**FIGURE 3:**
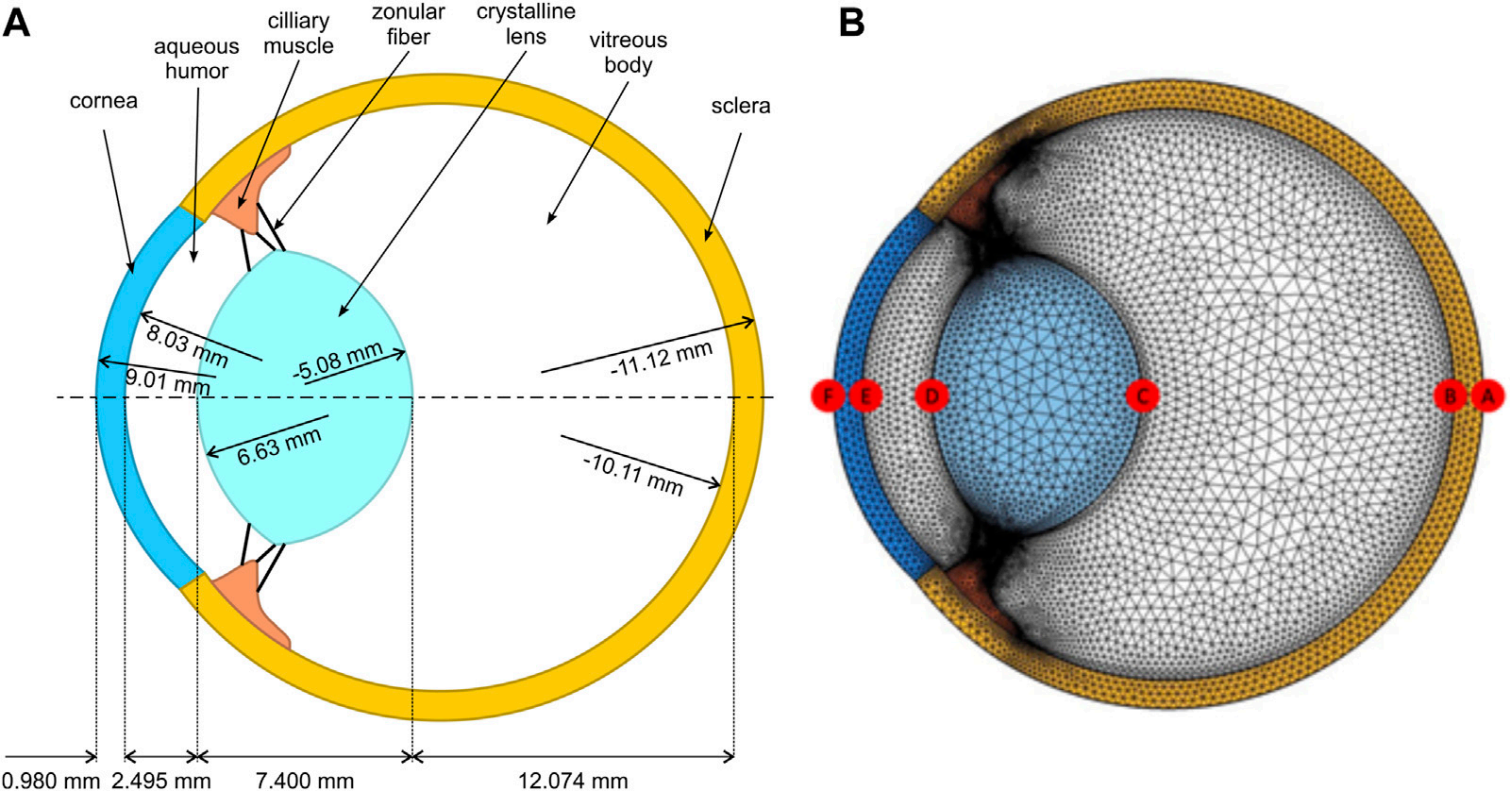
**(A)** Porcine eye model, including specific geometrical parameters with distances between surfaces and their corresponding radii of curvature. **(B)** 2D finite-element mesh showing the different points of interest.

In order to analyze the inertial motion of the crystalline lens, a numerical model was developed in COMSOL Multiphysics 6 using a porcine eye globe undergoing intraocular pressure as a fluid. Even though 3D models are accurate for some research, in this case, a 2D model is simpler, requires less computational effort, and still seems to be precise enough for analyzing the rotational motion of a planar model. The reason is that the movement of the eye followed by the lens rotation occurs in the same plane, and the other out-of-plane dimension can be neglected. This model contains the main and most influential components in eye biomechanics: lens, vitreous body, aqueous humor, zonular fibers, cornea, sclera, and ciliary muscle. The details of this generic porcine eye model (see Figure 3A) consist of geometrical dimensions that are based on the literature (Menduni et al., 2018; Regal et al., 2021). The layered structures of the choroid and retina were considered to be irrelevant for the simulations of the rotational motion due to their low thickness (Singh et al., 2017) and were ignored.

The crystalline lens is modeled to be suspended on 3 pairs of zonular ligaments, namely, anterior, equatorial, and posterior, with a thickness of 50 µm each. The attachment locations of the anterior and posterior zonules are 1.5 and 0.75 mm to the lens equator, respectively (Le, 2005; Wang et al., 2017).

#### 2.2.2 Material properties

Different constitutive material laws have been proposed in the literature for simulating the mechanical behavior of ocular tissues. They have been treated as pure linear elastic (Dai et al., 2017), hyperelastic (Lanchares et al., 2012), or viscoelastic (Kling et al., 2014). These models play a crucial role depending on what phenomena the model is focused on. For the purpose of this study, all constituents of the model were treated as linear elastic, isotropic, and homogeneous, with different material properties as reported in previous studies for a normal porcine eye. Table 1 presents the values of Young’s modulus, Poisson’s ratio, and density for the different porcine eye components.

**TABLE 1:** Material properties of the porcine eye (Watson et al., 2015; Wang et al., 2017).

Modeled parts	Young’s modulus [MPa]	Poisson’s ratio [-]	Density [kg/m^3^]
Sclera	28	0.49	1,400
Cornea	12	0.48	1,400
Ciliary muscle	11	0.45	1,600
Lens	1.5	0.49	1,100

Both the vitreous body and aqueous humor were modeled as viscous Newtonian incompressible fluid subjected to an initial pressure, which played the role of the average IOP magnitude in the model. The fluid–structure interaction (FSI) was considered to simulate the mechanical coupling between the humors and the solid structures of the eye. With a density of 1,000 kg/m^3^ and a dynamic viscosity of 0.00074 Pa⋅s, the fluid phase was considered viscous media (Singh et al., 2017).

#### 2.2.3 Mesh

Due to irregular geometry, triangular elements (Figure 3B) were used to discretize both the solid and fluid domains. In total, this model consisted of 48,139 elements, with an average element quality of 0.82. The element quality was measured using the built-in quality assessment (based on the equiangular skew) that provides a rating between 0 and 1 (Etminan et al., 2023). This optimum mesh size was selected after conducting a sensitivity analysis of the model. It was observed that reducing the global mesh size parameter in the program did not significantly affect the outcomes.

#### 2.2.4 Governing equations and boundary conditions

The time-dependent Navier–Stokes equations [Eq. (1)] govern the laminar flow around the eye structures. In the simulation, the fluid component initially has zero velocity, and the initial pressure is adjusted to correspond to the basic IOP of 15 mmHg (Sarmiento et al., 2023). The IOP has an important impact on the ocular structure, and it can increase or decrease in the cases where this parameter is associated with certain diseases. The IOP was applied to the inner surface of the sclera and the outer surface of the lens and ciliary body. Furthermore, the center of the sclera, serving as the pivot point for the eyeball's rotation, remains stationary without any linear movement. The time-dependent incompressible Navier–Stokes can be written as:

$$\rho \frac{\partial v}{\partial t}+\rho \nabla \cdot \left(v⊗v\right)-\mu \nabla^{2}v+\nabla p=\rho f,\nabla v=0$$ (1)

with **v** the velocity of the fluid, **p** the pressure, **f** the volumetric forces, ρ the density and µ the dynamic viscosity.

The multibody dynamics principles are employed to simulate the mechanical behavior of eye structures. It was also assumed that the shape and size of the sclera remains constant during motion. It underwent rotation (angular motion) but did not experience any deformation. The angular velocity of the eye during rotation was adjusted exactly the same as the one applied in the experimental setup. The multibody dynamics component involves modeling the mechanical behavior using the following equation:

$$\rho \frac{\partial^{2}u}{\partial t^{2}}=\nabla \cdot {\left(\text{FS}\right)}^{T}+\rho f,F=I+\nabla u$$ (2)

In Eq. (2), **u** is the displacement field, **F** is the deformation gradient tensor, **S** is the second Piola Kirchoff stress tensor and **I** is the identity matrix.

The dynamic interactions between the fluid and solid components are effectively captured through the implementation of a fully coupled FSI approach (Eq. (3)). Furthermore, a time-stepping scheme is used to ensure a synchronized update of the fluid and solid parameters throughout the simulation. The total force exerted on the solid boundary by the fluid in the spatial frame and the coupling between the velocities at the boundary can be written as:

$$f_{A}=\left[-pI+\left(\mu \left(\nabla v+{\left(\nabla v\right)}^{T}\right)-\frac{2}{3}\mu \left(\nabla \cdot v\right)I\right)\right]\cdot n,v=\frac{\partial u_{\text{solid}\;}}{\partial t}$$ (3)

Similarly, as in the *ex vivo* experiment, the eyeball rotated 90 degrees around its vertical axis (which is perpendicular to the plane of the 2D model), and all the mechanical displacement data were captured at every 4e-4 [s] during the movement for the apical points denoted as A–E specified in Figure 3B.

### 2.3 Optical simulations

To assess whether the mechanical model of the eye behaves like the real eye during the *ex vivo* experiment regarding the maximum overshooting amplitude, it was necessary to determine the relationship between the position of the Purkinje images and the actual alignment of the crystalline lens within the eye model. Since direct observation of the overshooting and inertial motion of the lens is not possible, the study focused on interpreting its optical effects through Purkinje imaging. In the final stages of the research methods, both tilt and lateral displacement, derived from mechanical simulations, were applied to obtain simulated Purkinje images.

**FIGURE 4:**
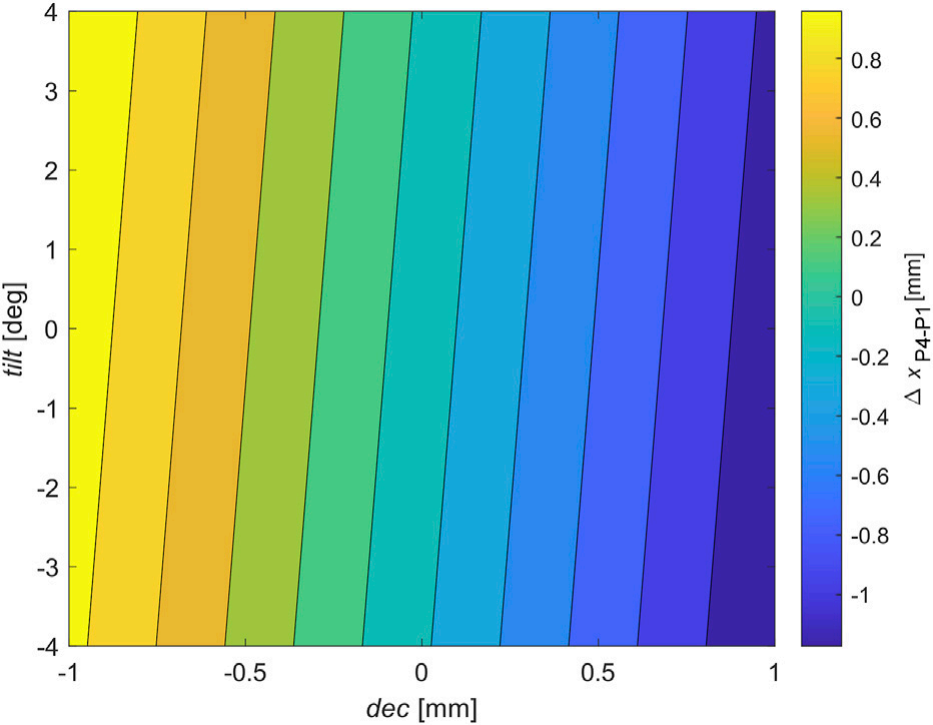
Resulting surface of Purkinje imaging optical modeling for the porcine eye model used in the study. The surface represents the approximation of the contribution of tilt (expressed in degrees) and decentration (expressed in millimeters) to the relative distance between P4 and P1 Purkinje images.

For this purpose, the computational procedure described by Boszczyk et al. (2023) was applied. Optical ray tracing simulations were performed in Zemax OpticStudio (Zemax, 2024). The porcine eye geometry described above was entered into the software application, as well as the values of the refractive indices for a wavelength of 850 nm of the cornea (1.3643), aqueous humor (1.3252), and lens (1.4617) (Wong et al., 2007; Sanchez et al., 2011), assuming these optical media to have optical dispersion similar to water. Then, *tilt* (lens rotation around the vertical axis) in the range from −4.0 to 4.0 deg and *dec* (lens decentration) from −1.0 to 1.0 mm were changed (*tilt* and *dec* magnitudes were given relative to the vertex of the anterior surface, point D shown in Figure 3B, of the lens). Purkinje images manifesting P1 and P4 were generated for each dataset of *tilt* and *dec*. The positions of the centers of the P1 and P4 images for each of the lens *tilt* and *dec* datasets were determined using image processing procedures in MATLAB described by Boszczyk et al. (2023). For the optical/geometrical parameters of the porcine eye model described above, the relation of the relative Purkinje distance *∆x_P4−P1_* as a function of *tilt* and *dec* can be approximated to a 3D plane (see Figure 4), given by the following equation (Eq. 4):

$$∆x_{P4-P1}=-0.000112+0.01977∙tilt-1.097∙dec,$$ (4)

where *∆x_P4−P1_* is expressed in millimeters, *tilt* is expressed in degrees, and *dec* is expressed in millimeters. The error of numerical simulation is represented by the constant term in Eq. 1 (Boszczyk et al., 2023). The goodness of fit of the plane to the optical simulation results, represented by $R^{2}$ , is 0.9999. This equation was used to estimate the Purkinje performance with the use of the lens alignment data simulated by means of FEM dynamic simulation, according to the workflow described by Boszczyk et al. (2023). Finally, the simulated Purkinje performance was compared to the experimental Purkinje performance *ex vivo* in order to estimate the quality of the computational model.

## 3 Results

**FIGURE 5:**
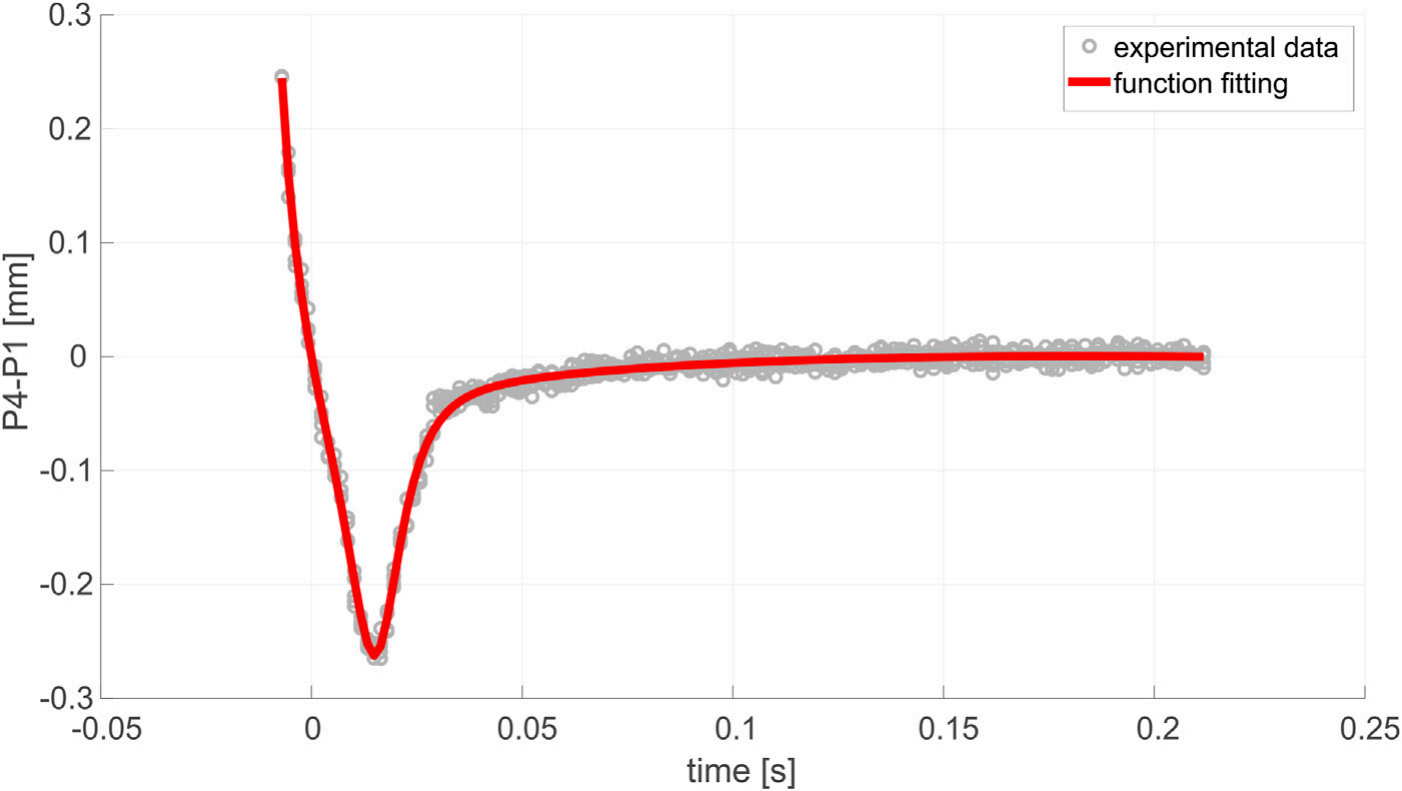
Overshooting effect observed in the examined eye *ex vivo*.

As mentioned, the porcine eye was tested *ex vivo*, and a precise description and characterization of lens overshooting was obtained using the high-speed recording of Purkinje images. The curve fitting of the experimental data, obtained by repeating the test four times, is presented in Figure 5.

**FIGURE 6:**
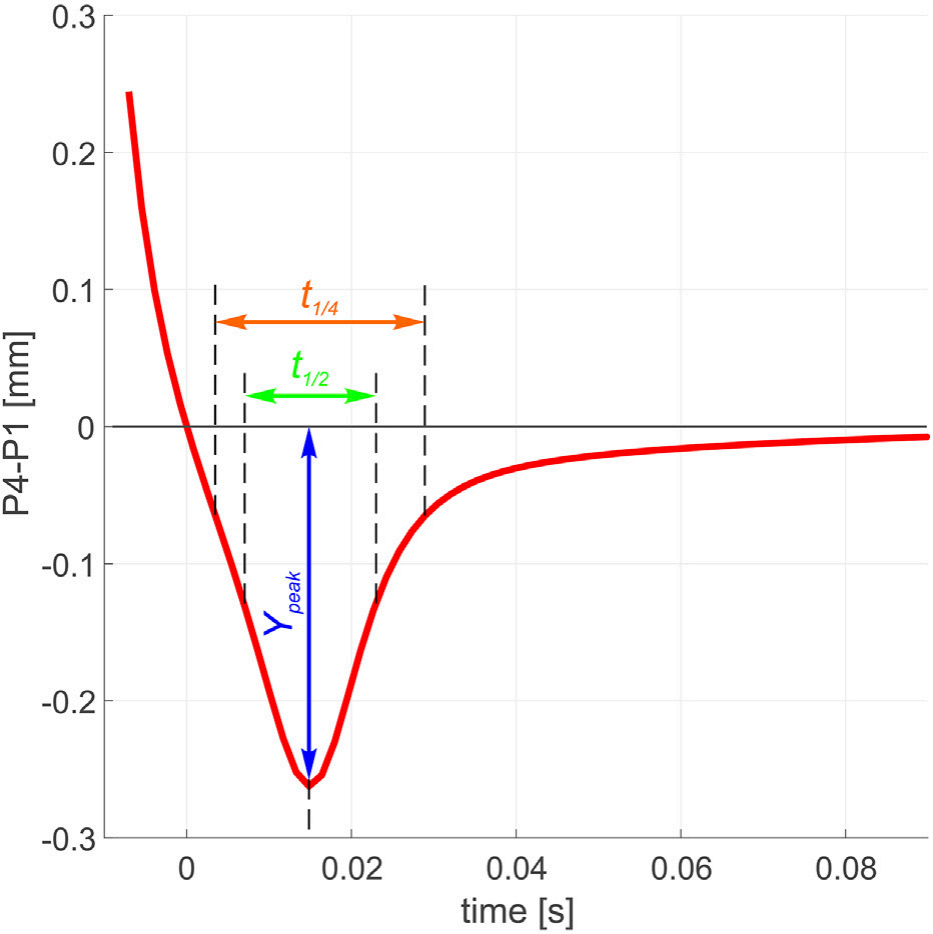
Parametrization of the overshooting trajectory.

Figure 6 shows a series of characteristic parameters in the evolution of P4–P1. *Y*_*Peak*_ is considered the maximum overshooting amplitude, while *t*_1/2_ and *t*_1/4_ are the width at half and quarter depths of the overshooting pattern, respectively.

In addition to the experimental analysis, a computer ray tracing simulation was applied in order to provide a better description of the physical sources of lens overshooting. Therefore, the fitting of the model based on the zonular Young’s modulus and damping ratio was performed against our experimental data.

**FIGURE 7:**
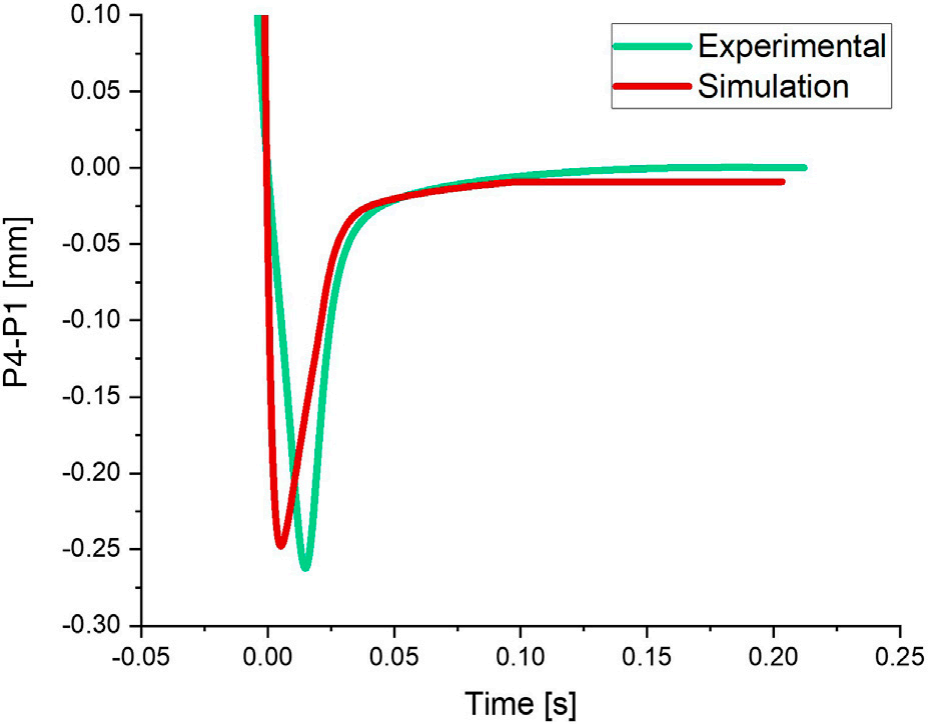
Overshooting magnitude in the experimental and simulation results.

Figure 7 shows changes in the location of the apex of the lens from the center of the cornea over time for experimental and modeling results. As can be observed, the maximum overshooting in the experimental result diagram is close to its equivalent in the modeling results.

The mechanical parameters, determined after conducting an optimization analysis, for the zonular fibers (Young’s modulus and Poisson’s ratio) are 950 and 0.49 [KPa], respectively.

The parameters characterizing the dynamic response of the lens (evolution of P4–P1) predicted by the model are shown in Table 2 together with those of the experimental test. As can be observed, the error is 13.92% for t_1/2_ [s], 5.50% for *Y*_*peak*_ [mm], and 1.59% for t_1/4_ [s], so it seems to be reasonably small.

**TABLE 2:** Detailed data related to the overshooting magnitude.

	*Y*_*peak*_ [mm]	Error (%)	t_1/2_ [s]	Error (%)	t_1/4_ [s]	Error (%)
Experimental	−0.2621	5.50	0.0158	13.92	0.0252	1.59
Simulation	−0.2477		0.018		0.0248	

In this section, the information obtained from the damping functions of the eye in the numerical model is reviewed and evaluated. Table 3 provides information about *t*_*balance*_ (stabilization time), *C*_*max*_ (maximum damping), *C*_*end*_ (damping at the end), *C*_*min*_ (minimum damping), Δ*C*, and Δ*C*_*max*._

**TABLE 3:** Damping data.

*t*_*balance*_ [s]	*C*_*max*_ [Pa⋅s]	*C*_*end*_ [Pa⋅s]	*C*_*min*_ [Pa⋅s]	*C*_*start*_ [Pa⋅s]	Δ*C* [Pa⋅s]	Δ*C*_*max*_ [Pa⋅s]
0.2	400	400	5	100	300	395

**FIGURE 8:**
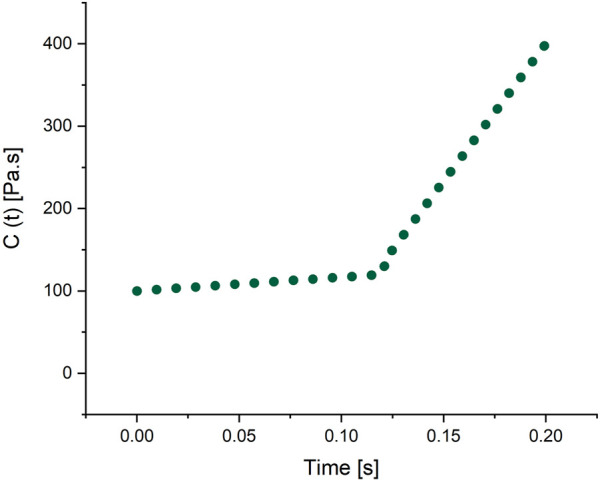
Damping function.

Figure 8 showcases a distinct pattern indicating a progressive increase in the damping function of the eye. An optimization of the damping factor function (using a range of trends and numbers for the damping factor) is shown in Figure 8 in order to have the displacement pattern mimic the experimental graph as closely as possible. The figure shows that damping increases smoothly until 0.125 s and then peaks as the lens confronts the most resistance when it hits its climax.

## 4 Discussion

As mentioned, the main purpose of this research was to model the dynamic behavior of the porcine eye and to determine the most influential factors on crystalline lens overshooting. For this reason, a porcine eye was analyzed, and experimental data were studied for modeling. Using this method, the most influential parameters on the overshooting phenomena can be obtained quantitatively. Based on the fact that optical and mechanical realizations have a strong physical background, simplicity, and compactivity, Purkinje imaging is still one of the most common imaging techniques used in order to investigate the activity and dynamics of oculomotor systems (Boszczyk et al., 2023).

The simulated effective Purkinje performance of lens overshooting is smooth and regular and is in good agreement with the P4–P1 pattern obtained in the *ex vivo* measurements, with comparable amplitude decay. Despite the fact that the displacement distribution depends on the rigidity of materials, such as ciliary muscle, zonular fibers, lens, cornea, sclera, and vitreous body, in our study, zonular mechanical properties were found to be the most influential parameters. Zonular fibers are responsible for suspending the crystalline lens on the ciliary muscle in mammalian eyes. These fibers transmit the forces exerted by the ciliary body to the lens during the accommodation process. Therefore, the elastic behavior of these bundles determines the efficiency of how the activity of the ciliary muscle is transferred to the crystalline lens (Bocskai et al., 2014). On the other hand, non-homogeneity of the mechanical properties among different structural requirements is likely to impact this matter. For example, during *ex vivo* tests, some zonular fibers retained their elasticity, indicating that these bundles lacked damping characteristics.

The compressibility of the zonule had a significant effect on the model. A value of 0.45 (i.e., slightly compressible) was used since the primary simulations showed that the model represented a greater general deformation and significant compression of the zonule. With a Poisson’s ratio of 0.49 (i.e., largely incompressible), the zonule showed significantly less deformation than for 0.45; therefore, the results are included in the simulations using both values. Hereafter, simulations performed using a Poisson’s ratio of 0.45 are referred to as the compressible model, while those with a value of 0.49 are referred to as the incompressible model (Bocskai et al., 2014). The wobbling behavior of the crystalline lens in the eye is significantly influenced by the compressibility and Poisson’s ratio of zonular fibers. The ability of the fibers to undergo deformation under pressure, known as compressibility, plays a role in their elastic response during dynamic changes in intraocular pressure and mechanical loading. If compressibility is excessive, it can compromise the stability of zonular fibers, leading to increased wobbling or displacement of the lens. On the other hand, Poisson’s ratio, which represents the transverse contraction of the material during stretching, affects the overall flexibility and adaptability of the zonular fibers. If an inappropriate choice of Poisson’s ratio is made, it can hinder the ability of the fibers to respond dynamically to changes in the mechanical environment, thereby impacting their role in maintaining optimal lens position and stability. The interplay between compressibility and Poisson’s ratio is crucial in governing the intricate biomechanics of zonular fibers and, consequently, in influencing lens wobbling within the eye.

An important aspect that needs to be addressed is the influence of the presence/lack of accommodation tension on the wobbling pattern, which was, for the first time, observed by Ehrmann et al. (2004). The phenomenon of eye accommodation is possible due to the activity of the ciliary muscle, which helps the eye change (adapt) the focus of the lens in order to see distant or near objects [7]. The changes in the crystalline lens curvatures are the basis of vision accommodation, which is agreed to be due to the contracting and relaxing of the ciliary muscles and zonules [28]. Prior prominent findings on this topic (Atchison, 1995; Charman, 2008; Lossing et al., 2012) associated the relaxed state of the accommodation mechanism with the ciliary muscle being in its loose state and its diameter being significantly larger. As a result, the tension of the zonular fibers is higher. The zonules make the crystalline lens stretch, and its overall diameter is higher as well. Under *ex vivo* conditions, the eye is considered fully unaccommodated, and the tension of the zonules transferred to the crystalline lens significantly attenuates the inertial motion of the lens during rotation. Therefore, unlike previous studies on wobbling conducted *in vivo* (Jacobi and Jagger, 1981; Tabernero and Artal, 2014), in our *ex vivo* experiment, instead of periodic oscillations, a pattern similar to the one shown in Figure 5 is manifested. The effect can be explained as follows: after sudden stopping, the lens shows some amount of overshooting; then, it gradually stabilizes due to the damping behavior of the zonules. However, it needs to be mentioned that our 2D model has some limitations that should be considered in future studies: turnover outflow at the trabecular meshwork (not the case in the current *ex vivo* model of the phenomenon), effects of the IOP magnitude, and the role of nonlinear mechanical models. Furthermore, the decision to focus on a single experiment was an intentional choice driven by the need to establish a preliminary understanding. The current study serves as a foundational exploration, so future research should indeed aim to incorporate a more extensive dataset to improve the consistency and generalizability of the findings.

## 5 Conclusion

In the present paper, an *ex vivo* porcine eye experiment to characterize lens overshooting was modeled using a Poisson’s ratio of 0.49 KPa and Young’s modulus of 950 KPa for the zonular fibers. These parameters were found to be the most influential on the time-varying damping of the system.

Based on the findings outlined in *Results*, which indicated a notable resemblance between the modeling and experimental data, it is viable to utilize this equation for *ex vivo* simulations of porcine eyes. This approach can effectively minimize the requirement for conducting a multitude of experiments.

## Data Availability

The original contributions presented in the study are included in the article/Supplementary Material; further inquiries can be directed to the corresponding author.

## Supplementary material

Supplementary Video 1
*Ex vivo* Purkinje video sequence.

Supplementary Video 2 Simulated Purkinje video sequence.

Supplementary Video 3 Animation presenting the lens inertial overshooting.

Supplementary Figure 1 Mechanical displacement magnitude.
