# Supplementary figures and images for: Investigation of crystalline lens overshooting: ex vivo experiment and optomechanical simulation results
Source: Front Bioeng Biotechnol. 2024 Apr 9;12:1348774. doi: 10.3389/fbioe.2024.1348774 (PMC11035874; doi:10.3389/fbioe.2024.1348774)

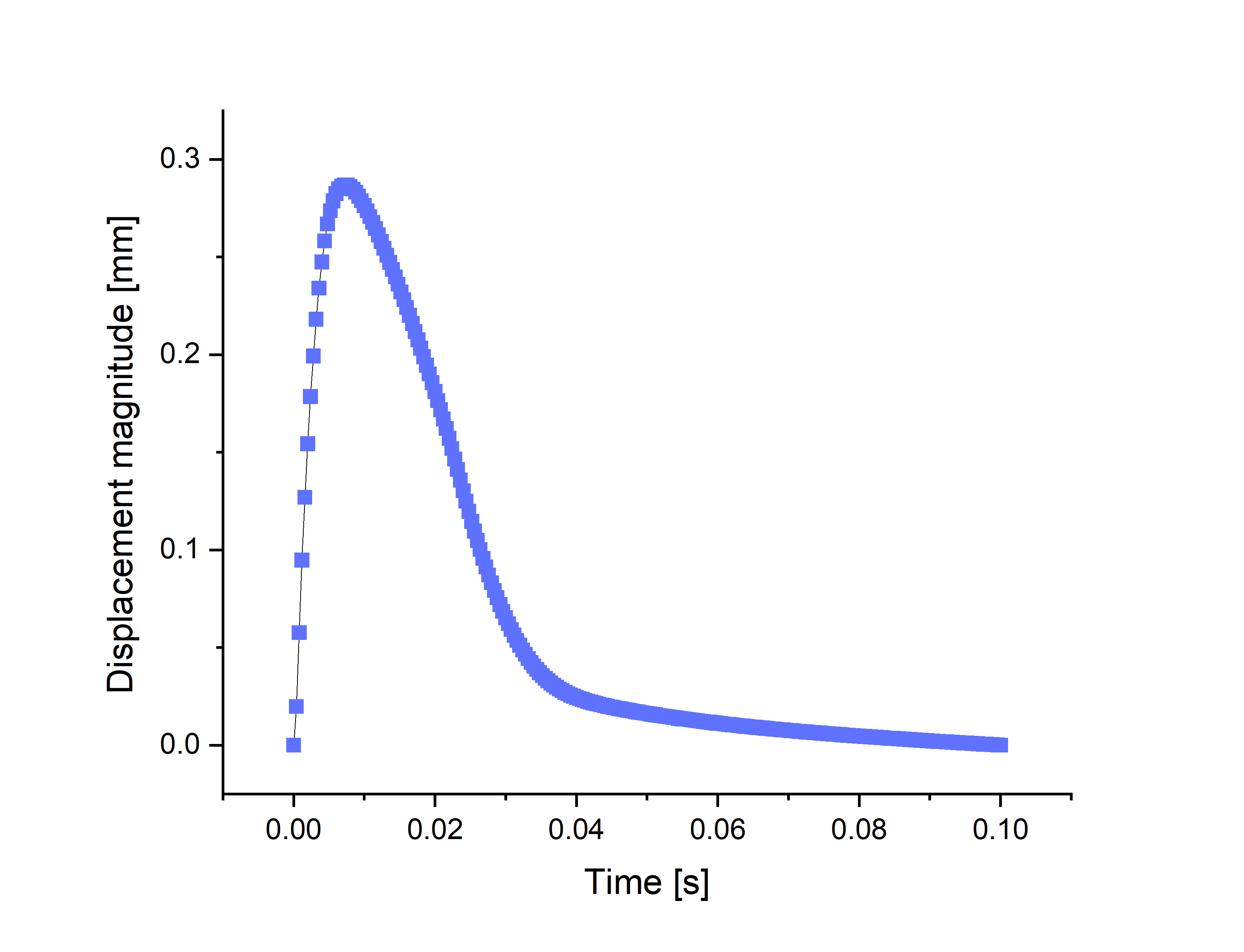

Supplement: Supplementary file 3 [file Image1.JPEG]
