# Supplementary material for: Investigation of crystalline lens overshooting: ex vivo experiment and optomechanical simulation results
Source: Front Bioeng Biotechnol. 2024 Apr 9;12:1348774. doi: 10.3389/fbioe.2024.1348774 (PMC11035874; doi:10.3389/fbioe.2024.1348774)
Supplement: Supplementary file 4 [file DataSheet1.PDF]

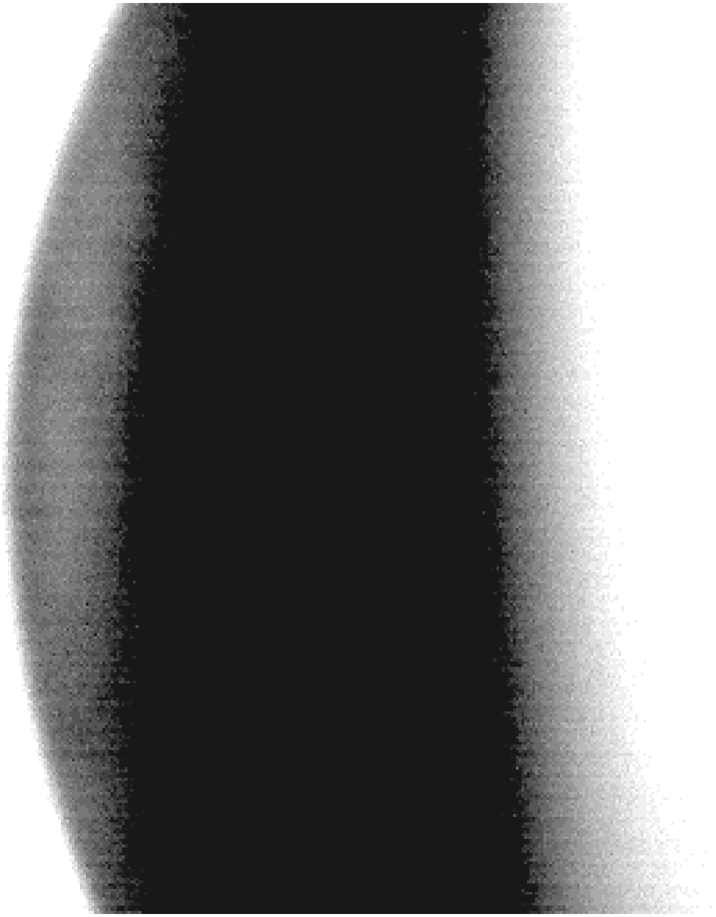

Visualization 1. Ex vivo Purkinje image.

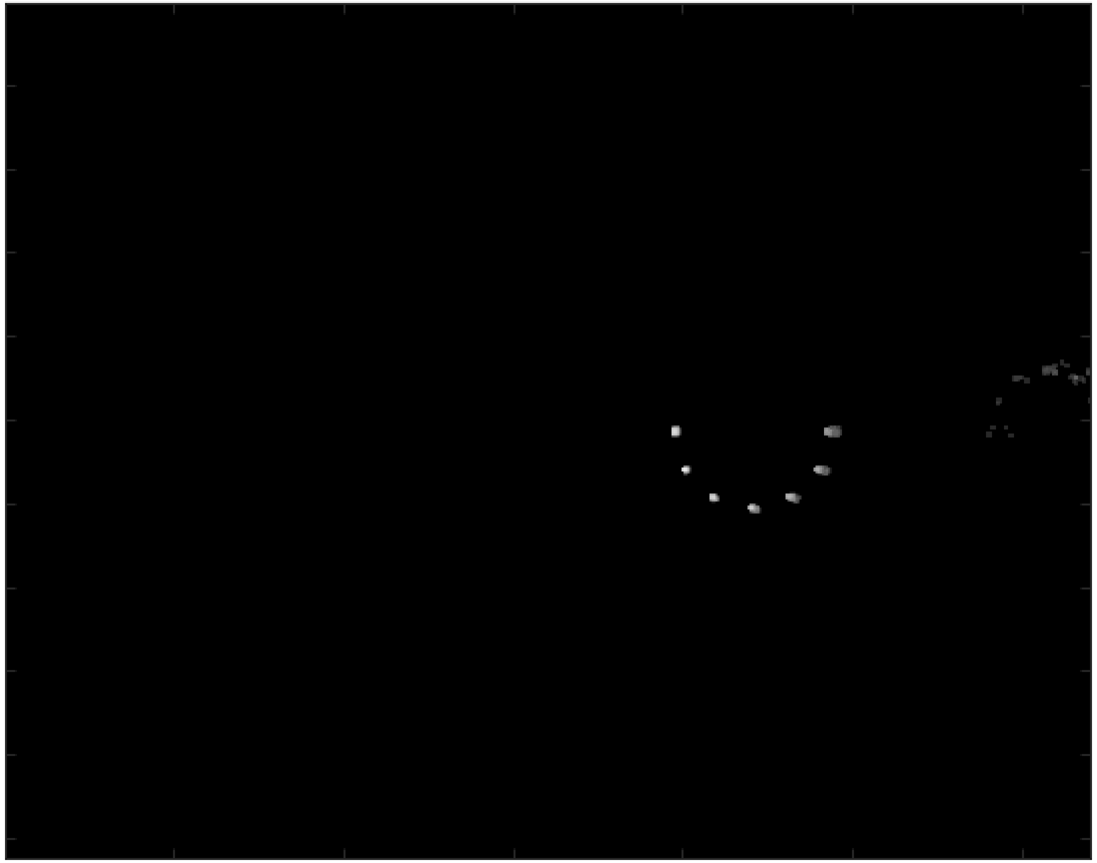

Visualization 2. Simulated Purkinje image.

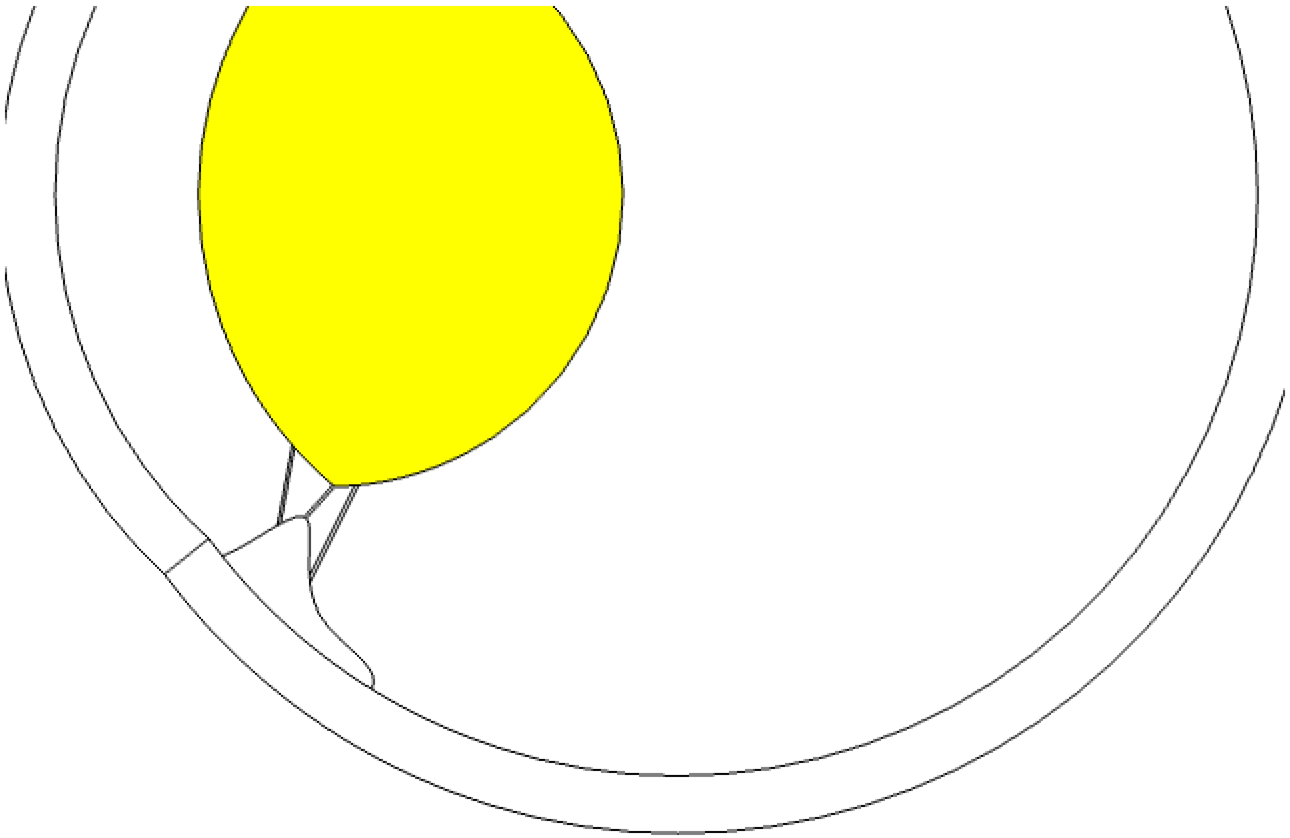

Visualization 3. Lens overshooting.

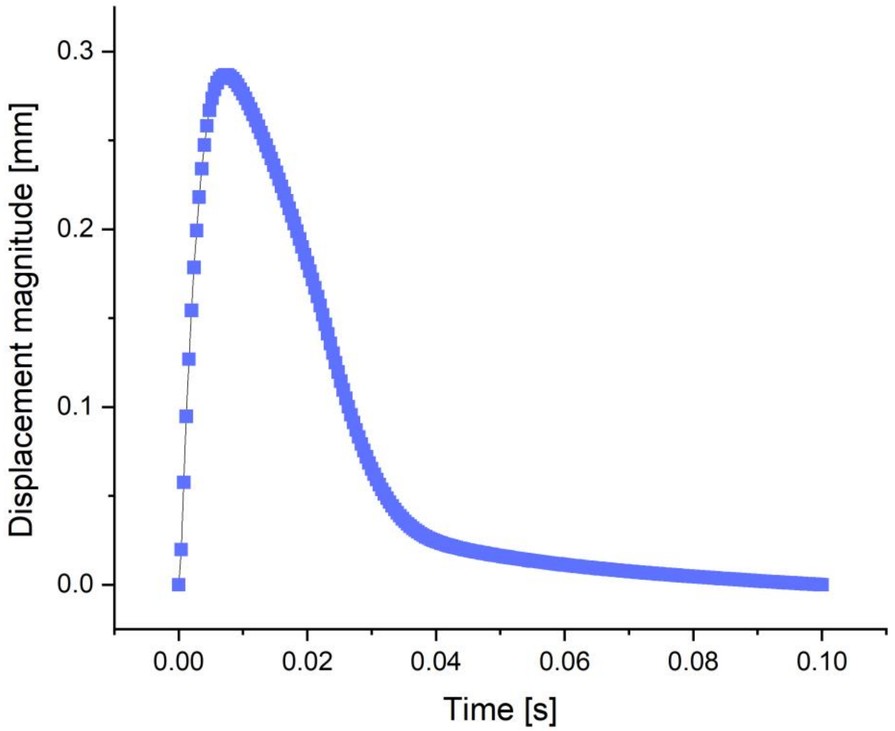

Visualization 4. Mechanical displacement magnitude.
